# Decoupling of Friction and Solid Fraction in Dense Granular Flow Across Six Orders of Magnitude of Shear Rate

**DOI:** 10.3390/ma19183920

**Published:** 2026-09-15

**Authors:** Yi Zhang, Yangshuai Zheng, Hui Luo, Xin Gao

**Affiliations:** 1 College of Intelligent Construction and Manufacturing, Sichuan Technology and Business University, Chengdu 611745, China; 2 State Key Laboratory of Geo-Hazard Prevention and Geo-Environment Protection, Chengdu University of Technology, Chengdu 610059, China; 3 The 2nd Geological Brigade of Sichuan, Chengdu 610041, China

**Keywords:** dense granular flow, shear rate, μ(I) rheology, solid volume fraction, shear localization, discrete-element method

## Abstract

The μ(I) framework for dense granular flows treats the macroscopic friction (μ), defined as the ratio of shear to normal stress transmitted across the sheared assembly, and the solid volume fraction (φ) as single-valued functions of the inertial number (I). To test if these responses evolve together across shear rates, we used 3D discrete-element simulations of ring shear on a monodisperse assembly of spherical glass beads (diameter d = 1 mm), confined between two parallel toothed platens at 100 kPa normal stress. Shear velocity was varied over six orders of magnitude (10^−4^ to 10 m/s), spanning a nominal inertial number I ≈ 10^−6^ to 10^−1^. Throughout, I denotes a bulk value defined from the imposed shear rate v/H and the applied normal stress; the local inertial number varies with height and is reported separately. We found that steady-state friction closely follows the *μ*(*I*) law, rising monotonically with rate. In contrast, bulk solid fraction *φ* is non-monotonic: it falls as rate increases, reaches a minimum near *I* ≈ 10^−3^, and recovers at higher rates, making a single *φ*(*I*) curve a poor fit. Height-resolved fields explain this decoupling. At higher rates, shear concentrates near the driving boundary, and strong vertical agitation drives cross-layer mixing that re-compacts the lower bed. Consequently, bulk *φ* averages a dilating top with a re-densifying base, losing its one-to-one link to *I*. Instead, over the conditions tested, the contact density (n_c_) decreases monotonically—by the same factor whether it is normalised per unit height, per unit volume, or per particle—and so remains in step with the friction where the bulk solid fraction does not. The contact-force distribution broadens, and the fabric anisotropy grows as the flow becomes inertial, so that load is carried through fewer, more strongly oriented chains. These results show that friction and packing can respond to shear rate on different terms, and that bulk *φ* should be used with care as a state variable in rate-dependent dense flows.

## 1. Introduction

Dense granular materials flow in ways that lie between those of a solid and a liquid [1,2]. When sheared, these materials deform through particle rearrangements, frictional sliding, and the repeated build-up and collapse of force-chain networks, rather than through smooth internal strain. This grain-scale activity makes their bulk behaviour strongly nonlinear and history-dependent, and it underlies many natural and industrial problems, from landslides and fault slip to powder handling and silo discharge [3,4]. Predicting how such materials deform therefore requires linking the macroscopic response to the underlying microstructure.

The state of a sheared granular flow is captured compactly by the dimensionless inertial number, $I=\overset{\cdot }{\gamma }d/\sqrt{P/\rho }$ where $\overset{\cdot }{\gamma }$ is the shear rate, d the grain diameter, ρ the grain density, and P the confining pressure [5,6]. The inertial number measures the competition between grain inertia and the confining pressure. At low I (below about 10^−3^), lasting frictional contacts dominate, and the flow is quasi-static and nearly rate-independent. At intermediate I (10^−3^ to 10^−1^), inertial and frictional effects coexist in the dense flow regime, where the μ(I) rheology applies. At high I, collisions take over, and the material dilutes [3,7]. This progression from enduring, force-chain-dominated contacts to collisional dissipation underpins constitutive descriptions that bridge the quasi-static and rapid-flow regimes [8,9]. The μ(I) framework writes the macroscopic friction μ = τ/P—an internal property of the granular assembly, given by the shear stress τ it transmits divided by the normal stress P acting on it, and not a wall-friction coefficient between the grains and any external surface—and the solid volume fraction φ as smooth, single-valued functions of I, and it has been tested across many geometries and materials [10,11]. It has also been adopted as the constitutive core of continuum models for geophysical mass flows such as avalanches and landslide runout [12], so that any breakdown of the φ(I) closure bears directly on these applications.

A central assumption of this framework is that μ and φ are two faces of the same state that both follow once I is fixed, which holds well in homogeneous, steady shear. The framework is local, however, and real flows are often far from homogeneous [11,13]. When a strain-rate gradient is present, the local μ(I) relation can fail, which has motivated a family of nonlocal models that add an intrinsic length scale set by the grain size [14,15,16]. In a confined shear cell, the flow is strongly stratified: shear and dilation concentrate near the driving boundary, while the far field creeps or stays still [17,18,19]; within such localised bands, the contact fabric reorganises toward an anisotropic critical state [20]. Under these conditions, a single bulk-averaged value need not represent the state of every layer, and μ and φ need not track I in the same way. Whether this coupling actually holds across a wide range of shear rates has rarely been tested directly.

Most studies of rate effects have used a narrow velocity window and have examined individual flow quantities in isolation [21,22,23]. The microstructural picture is clearer for stress effects than for rate effects: raising the confining stress is known to densify the contact network and broaden the shear zone, whereas the way shear rate reshapes localization, dilatancy, particle kinematics, and force-chain topology together—over many decades of rate—has not been mapped within a single system. Discrete-element studies of dry monodisperse assemblies have mapped steady-state loci that link the stress ratio and the solid fraction to the shear intensity [24], yet these loci are typically established over a limited range and treat φ as single-valued in I. In particular, it is not established whether the macroscopic friction and the packing fraction respond to shear rate in the same way. Nor is it clear which microstructural measure, if any, remains a reliable order parameter once the bulk packing ceases to be one.

Here we address these questions with three-dimensional DEM simulations of annular shear. We hold the normal stress fixed at 100 kPa and vary the shear velocity over six orders of magnitude (10^−4^ to 10 m/s), covering I ≈ 10^−6^ to 10^−1^. As functions of rate, we resolve the steady-state friction and axial dilation, the height profiles of velocity and velocity fluctuation, the solid-fraction field, individual particle trajectories and vertical agitation, and the force-chain network. The central result is a clean decoupling: the friction follows μ(I) closely, but the bulk solid fraction is non-monotonic and departs from any single φ(I) curve. We trace this decoupling to rate-driven localization and cross-layer mixing, and we identify the contact density as the structural measure that stays well-behaved when bulk φ does not.

## 2. Materials and Methods

### 2.1. Model Geometry and Boundary Conditions

**Figure 1:**
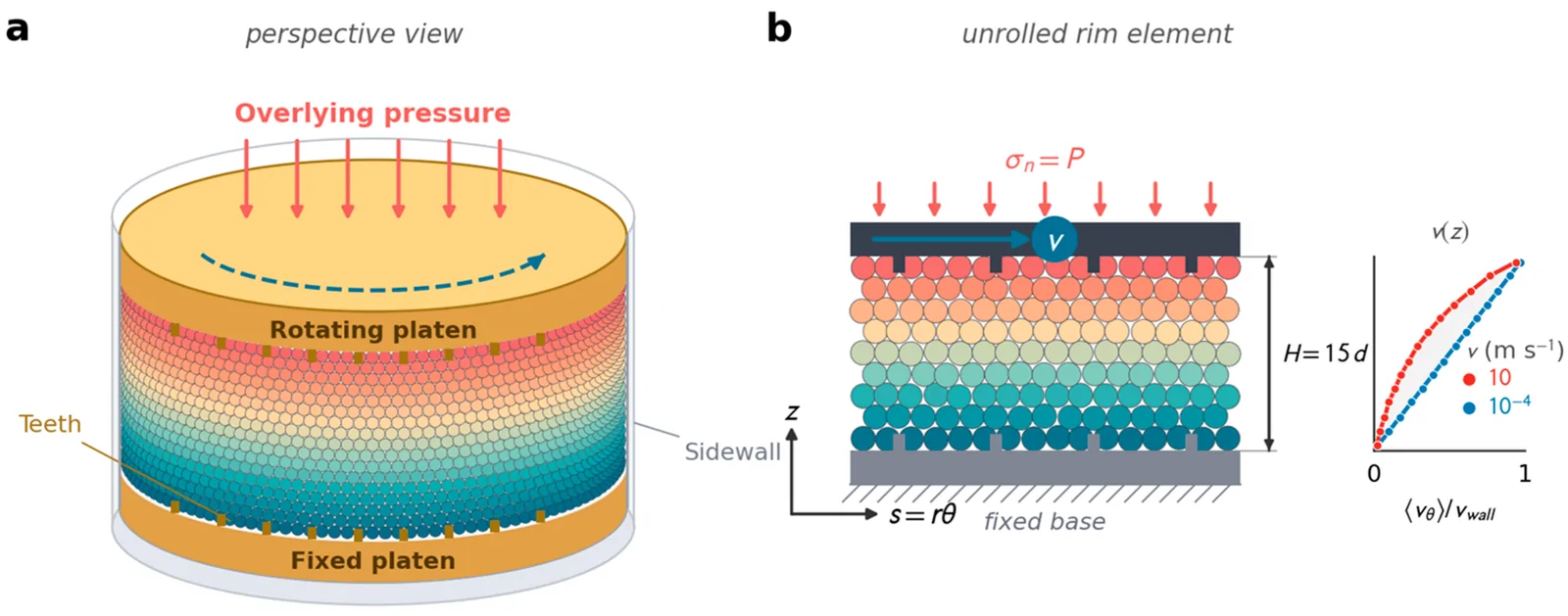
Ring-shear model. (**a**) Perspective view of the annular cell: a monodisperse glass-bead column (coloured by height) confined between a fixed and a rotating toothed platen, with a rigid frictionless sidewall; the top platen imposes a constant normal stress and rotates to shear the bed. (**b**) Unrolled rim element (s = rθ, z) showing the normal stress, driving velocity v, and steady-state velocity profiles ⟨v_θ_⟩/v_wall_ at v = 10^−4^ and 10 m/s.

The confined ring-shear flow was simulated with a three-dimensional discrete-element method (DEM), which treats grains as nonlinear elastic spheres carrying translational and rotational degrees of freedom; the contact laws are detailed in Section 2.2 and follow formulations developed and validated in earlier studies [25]. The model reproduces an annular (ring-shear) cell, which sustains continuous, steady shear without the displacement limits and end effects of direct-shear or triaxial tests (Figure 1). The cell is a right circular cylinder of outer radius R = 20 mm. The granular column fills the entire cross-section rather than an annular gap: the term “ring shear” refers to the rotational (torsional) driving, which allows unlimited shear displacement, and not to a ring-shaped sample. A monodisperse assembly of glass spheres of diameter d = 1 mm and density ρ = 2500 kg/m^3^ fills the cell to an initial height H = 15 mm (=15d). The column is deliberately tall so that the full vertical structure of the flow—from the active layer near the driving boundary to the slow base—can be resolved [26].

The assembly is confined between a fixed bottom platen and a rotating top platen. Both platens carry explicitly modelled teeth of 1 mm height, equal to one grain diameter, rather than an imposed no-slip condition; the grains interact with these teeth through the same contact law used between grains. A roughness of about one grain diameter is the standard choice in laboratory ring-shear cells, where the platens are usually roughened with glued grains of the tested material, and it is sufficient here to suppress wall slip. A rigid, frictionless outer wall confines the grains radially while allowing free tangential motion. The top platen turns at a prescribed angular velocity, set so that the tangential speed at the outer radius equals the target value v; its height is free and is controlled by a numerical servo algorithm—not by an experimental loading frame—which adjusts the platen position at every time step so that the normal stress measured on the platen is held at σ = 100 kPa. The platen is given a translational degree of freedom in z only, and no artificial damping is applied to it. The bed can therefore dilate or compact while the confining stress stays constant [5,6]. The tangential velocity in an annular cell increases with radius, so the local shear rate is not uniform across the cell. To keep the local kinematics close to simple plane shear, the velocity, velocity-fluctuation, trajectory, and contact-network statistics are evaluated within a narrow outer band one grain diameter wide (r = 19–20 mm), across which the radial variation in shear rate is negligible [17,18]. This assumption is verified quantitatively in Section 3.3 (Figure A2), where we show that the horizontal plane is close to rigid-body rotation and that the vertical localization is reproduced at every radius. The height-resolved solid-fraction profile, which needs a larger sample for stable layer statistics, is instead computed over the wider band r = 5–20 mm, excluding only the slow near-axis core; the bulk solid fraction is obtained separately from the top-platen displacement (Section 2.3).

### 2.2. Contact Model and Material Parameters

Grain interactions follow the Hertz–Mindlin law, which combines nonlinear elastic contact in the normal direction with Coulomb friction in the tangential direction [27,28,29]. For a contact between grains i and j, the normal force combines a Hertzian elastic term with a viscous damping term,

(1) $$F_{n}=4/3\;E^{*}\sqrt{R^{*}}\;\delta_{n}^{3/2}\;-\;2\sqrt{5/6}\;\beta \sqrt{S_{n}\;m^{*}}\;v_{n}^{\;rel},\;\;S_{n}=2E^{*}\sqrt{R^{*}\delta_{n}},$$

where $\delta_{n}$ is the normal overlap, $v_{n}^{\;rel}$ the normal component of the relative velocity, and $S_{n}$ the normal stiffness. The tangential force is

(2) $$F_{t}=-\;S_{t}\;\delta_{t}\;-\;2\sqrt{5/6}\;\beta \sqrt{S_{t}\;m^{*}}\;v_{t}^{\;rel},\;\;S_{t}=8G^{*}\sqrt{R^{*}\delta_{n}},$$

with tangential overlap $\delta_{t}$, relative tangential velocity $v_{t}^{\;rel}$, and tangential stiffness $S_{t}$. The tangential force grows elastically until it reaches the Coulomb limit $|F_{t}|\leq \mu_{p}F_{n}$, beyond which the contact slides. The effective Young’s modulus, shear modulus, radius, and mass are

(3) $$\frac{1}{E^{*}}=\frac{1-\nu_{i}^{2}}{E_{i}}+\frac{1-\nu_{j}^{2}}{E_{j}},\;\;\frac{1}{G^{*}}=\frac{2-\nu_{i}}{G_{i}}+\frac{2-\nu_{j}}{G_{j}},$$

(4) $$\frac{1}{R^{*}}=\frac{1}{R_{i}}+\frac{1}{R_{j}},\;\;\frac{1}{m^{*}}=\frac{1}{m_{i}}+\frac{1}{m_{j}},$$

and the damping coefficient is set by the restitution coefficient e through

(5) $$\beta =\frac{-lne}{\sqrt{{ln}^{2}e+\pi^{2}}}.$$

Rolling resistance is represented by a constant torque that opposes the relative rotation at the contact,

(6) $$M=-\;\mu_{r}\;F_{n}\;R_{r}\;\frac{\omega_{ij}}{\left|\omega_{ij}\right|},$$

where $\omega_{ij}$ is the relative angular velocity of the contacting grains, $R_{r}$ the rolling radius, and $\mu_{r}$ the rolling-friction coefficient. This constant-torque form represents the angularity and surface roughness of real grains that ideal spheres lack [30,31,32]. Energy loss in collisions is controlled through the restitution coefficient e=0.938 [33].

The grains are modelled as soda–lime glass with a shear modulus G=30 GPa and Poisson’s ratio ν=0.2, giving a Young’s modulus $E=2G\left(1+\nu \right)=72$ GPa. The interparticle sliding friction is $\mu_{p}=0.9$, within the range reported for glass–glass contacts under shear, and the rolling friction is 0.1; the particle–wall sliding and rolling frictions are 0.04 [34]. All parameters are listed in Table 1. All DEM simulations in this study were performed with LIGGGHTS-PUBLIC, an open-source discrete element method package (https://github.com/CFDEMproject/LIGGGHTS-PUBLIC, accessed on 13 September 2026) , using the Hertz–Mindlin contact model with the constant-torque rolling-resistance extension described above. Motion is integrated with an explicit Euler scheme using a time step $\Delta t=0.2\;t_{R}$, where $t_{R}$ is the Rayleigh time, the period for an elastic wave to cross one grain [25]. This step resolves the contact response and keeps the integration stable.

### 2.3. Loading Protocol and Results Processing

**Figure 2:**
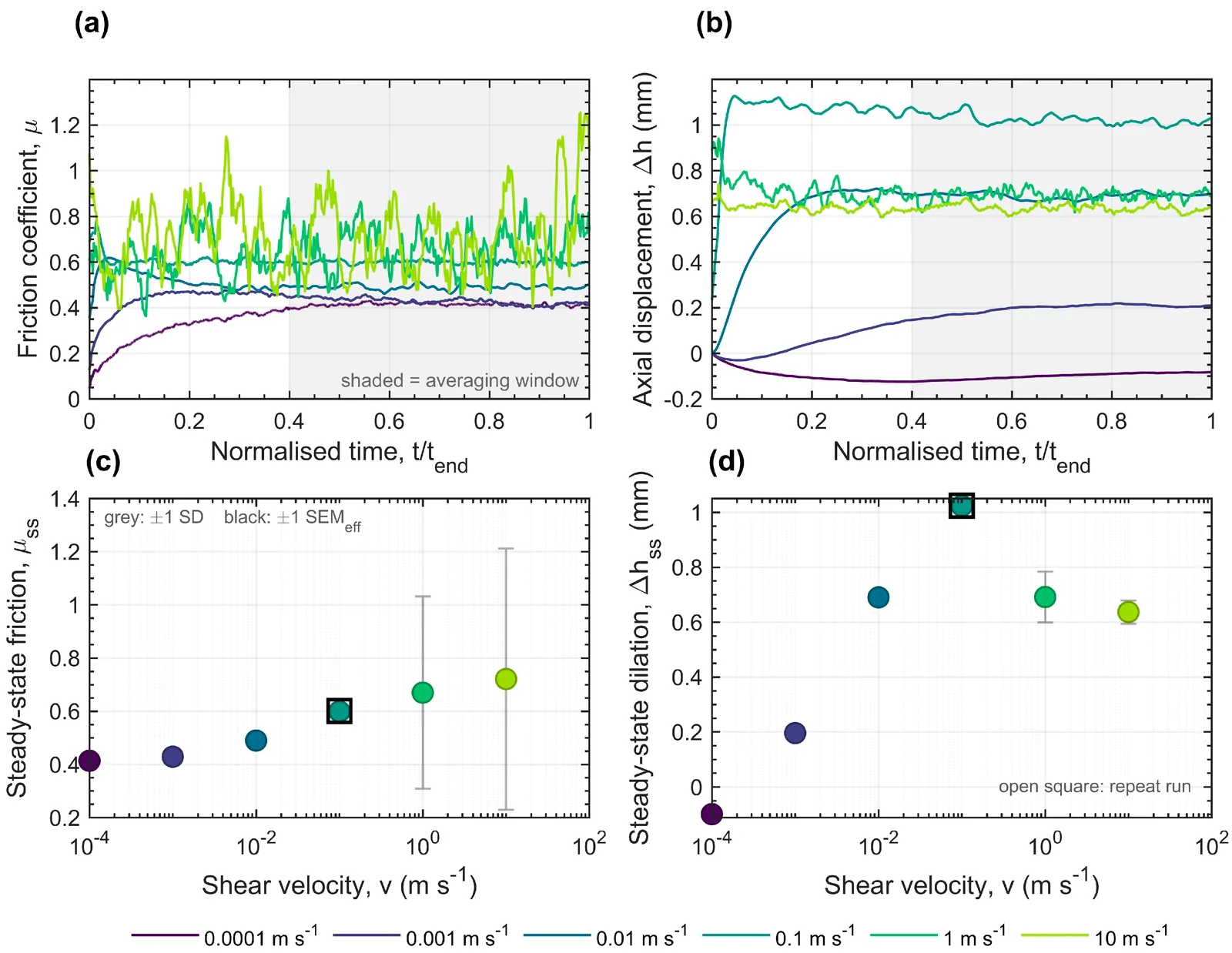
Macroscopic response at σ = 100 kPa for shear velocities v = 10^−4^–10 m s^−1^. (**a**) Friction coefficient and (**b**) axial displacement versus normalised time; the shaded band marks the averaging window (the final 60% of the run) over which all steady-state values are computed. (**c**) Steady-state friction μ_ss_ and (**d**) steady-state dilation Δh_ss_ versus shear velocity. Error bars show ±1 standard deviation (grey) and ±1 standard error of the mean corrected for temporal correlation (black), as defined in Section 2.3 and tabulated in Table 2. μ_ss_ rises monotonically, whereas Δh_ss_ peaks near v = 10^−1^ m s^−1^. Open squares mark an independent repeat run at v = 10^−1^ m s^−1^, which reproduces the original. Colour encodes shear velocity on a perceptually uniform sequential scale.

**Figure 3:**
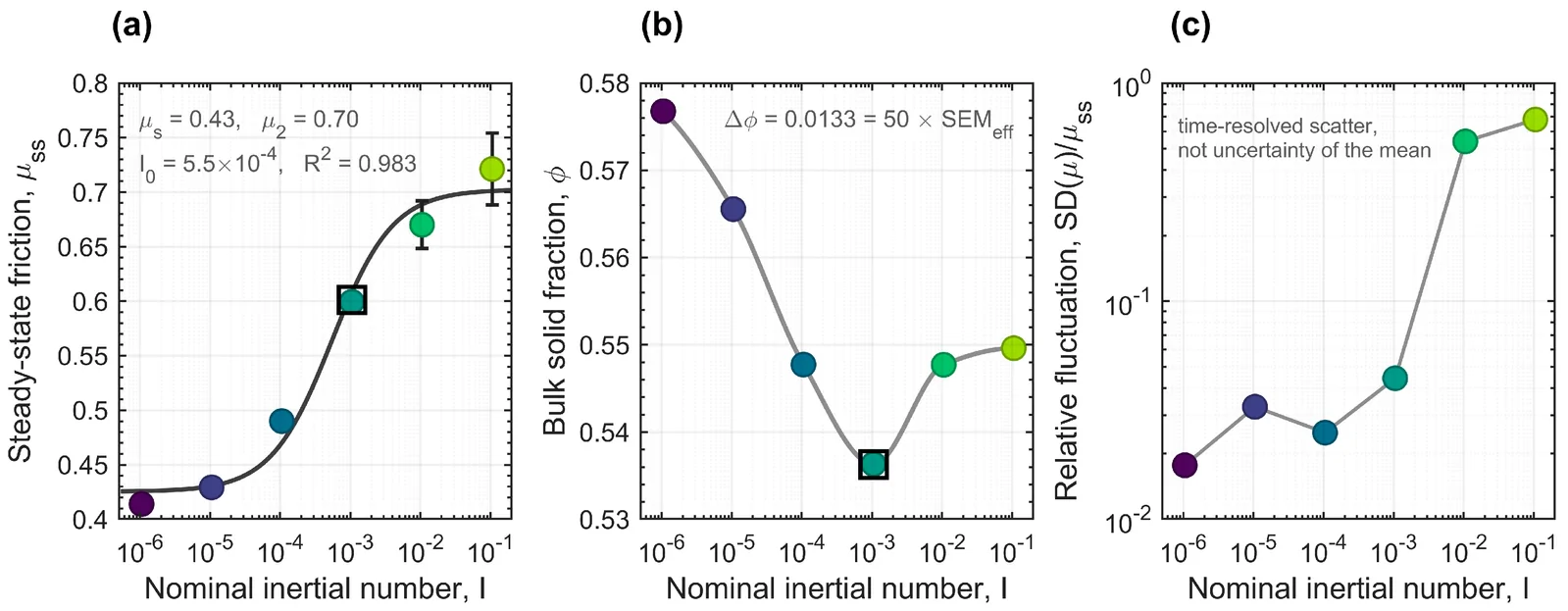
Steady-state rheology versus the nominal inertial number I. (**a**) Friction μ_ss_ with the μ(I) fit μ(I) = μ_s_ + (μ_2_ − μ_s_)/(I_0_/I + 1) (μ_s_ = 0.43, μ_2_ = 0.70, I_0_ = 5.5 × 10^−4^, R^2^ = 0.98). (**b**) Bulk solid volume fraction φ; the grey curve traces the measured trend, which passes through a minimum near I ≈ 10^−3^ and cannot be represented by a single monotonic φ(I) law. (**c**) Relative amplitude of the friction fluctuations, SD(μ)/μ_ss_, which grows by a factor of about 38 across the sweep. Error bars in (**a**,**b**) show ±1 standard error of the mean corrected for temporal correlation; the corresponding standard deviations are given in Figure 2 and Table 2. Open squares mark an independent repeat run at v = 10^−1^ m s^−1^.

**Figure A1:**
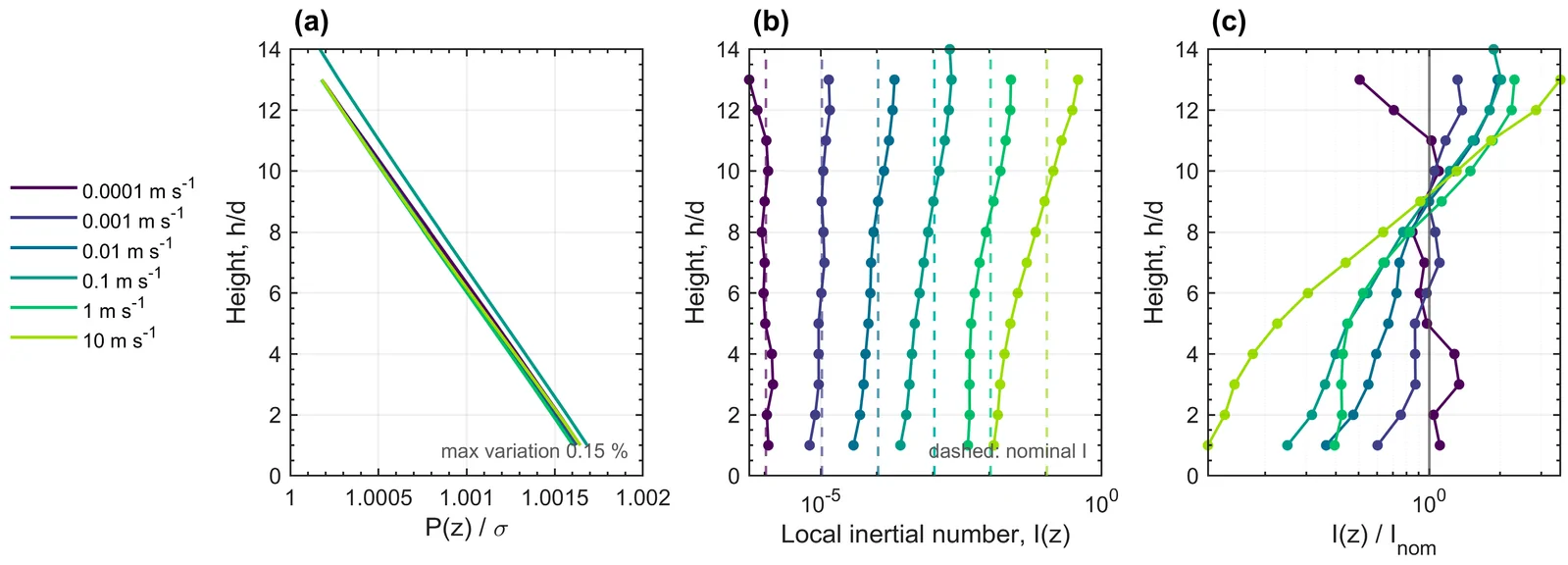
Local inertial number resolved with height, in the outer band (r = 19–20 mm). (**a**) Pressure profile P(z) normalised by the applied normal stress σ; the grain self-weight changes P by less than 0.2% over the whole bed. (**b**) Local inertial number I(z) for the six shear velocities; dashed vertical lines mark the corresponding nominal values I = (v/H)d√(ρ/σ). (**c**) Ratio I(z)/I_nom_; the solid vertical line marks unity.

Six cases were run at shear velocities v = 10^−4^, 10^−3^, 10^−2^, 10^−1^, 1, and 10 m s^−1^, all at σ = 100 kPa. Using the bulk shear rate $\overset{\cdot }{\gamma }$ = v/H, the nominal inertial number ranges from about 1.1 × 10^−6^ at the slowest rate to 1.1 × 10^−1^ at the fastest, so the sweep covers the deep quasi-static regime up to the edge of the inertial regime. This nominal I is a bulk value set by the boundary conditions; the local inertial number varies with height, because both the shear rate and the pressure change through the bed. The size of that variation is quantified in Section 3.2 (Figure A1), and all values of I quoted below are nominal unless stated otherwise. To check that the results are reproducible, the case at v = 0.1 m s^−1^—which sits at the minimum of the bulk solid fraction and therefore anchors the central finding—was run a second time from an independently generated initial packing; the two realisations are compared directly in Figure 2 and Figure 3.

In each case, the assembly was first consolidated under the target normal stress for 0.5 s to reach mechanical equilibrium, then sheared until μ and the axial displacement Δh reached statistically steady values. Particle positions, velocities, and contact forces were recorded at fixed intervals, and all steady-state quantities were averaged over the final 60% of the shear (t/t_shear_ ≥ 0.4), the interval over which μ and Δh fluctuate about constant means with no residual drift. All steady-state quantities are reported as the mean over this window, computed from the raw (unsmoothed) time series. Two uncertainty measures accompany them: the standard deviation SD, which describes the amplitude of the physical fluctuations, and the standard error of the mean. Because these time series are strongly autocorrelated, the standard error is computed from an effective sample size N_eff_ = N/(2τ_int_), where τ_int_ is the integrated autocorrelation time of the series; using the raw sample count would understate the uncertainty by up to an order of magnitude. Table 2 lists both measures for every case, together with τ_int_, N_eff_, the duration of the averaging window and the shear strain accumulated within it.

For the height-resolved velocity and its fluctuation, the outer band (*r* = 19–20 mm) was divided into horizontal layers one grain diameter thick, and within each layer we computed the mean tangential velocity and the velocity fluctuation (the standard deviation of particle velocities, a proxy for granular temperature; [35,36]). The height-resolved solid-fraction profile φ(*z*) was evaluated on the wider band (*r* = 5–20 mm) using the same one-diameter layers, with each grain’s contribution to a layer taken as the exact spherical-cap volume it occupies within that layer, so that grains straddling a boundary are partitioned correctly.

The macroscopic friction μ was taken as the ratio of tangential to normal force on the top platen. The bulk solid fraction was obtained from the total grain volume and the instantaneous bed volume set by the servo-controlled top-platen height; this whole-bed measure, rather than any radial sub-sample, is the φ reported in Figure 2 and Figure 3. Individual particles in the outer band were tracked through the steady state to follow vertical migration and to measure the vertical fluctuation σ_z_. For each tracked grain, σ_z_ is the standard deviation of its height over the same steady-state window used for all other averages, namely the final 60% of the shearing stage. In absolute terms, this window spans 28.0 s at v = 10^−4^ m s^−1^ and 2.7 s at v = 10 m s^−1^, corresponding to accumulated shear strains of 0.19 and 1800, respectively. For the contact network in the same band, normal forces in each layer were normalised by the layer mean, and contacts above 1.5 ⟨F_n_⟩ were classed as strong chains [37,38]. We also computed the fabric anisotropy *a* from the distribution of contact orientations [39], the strong-chain fraction *r*_strong_, and the contact density *n*_c_ (the number of contacts per unit height), which together describe how the load-bearing structure changes with rate.

## 3. Results

### 3.1. Friction Coefficient and Axial Dilation

Figure 2 shows the macroscopic response at σ = 100 kPa. In every case, the friction coefficient rises through a short transient and then fluctuates about a constant mean for the remainder of the run (Figure 2a), and it is over the final 60% of the shearing stage—the shaded band in Figure 2a,b—that all steady-state values are computed. What changes markedly with rate is the character of that steady state rather than its existence. At the lowest velocity, the signal is smooth: the standard deviation of μ about its mean is 0.007, or 1.8% of the mean. The fluctuations grow steadily with rate until, at v = 10 m s^−1^, they reach 0.49, some 68% of the mean, with individual excursions exceeding unity. The mean itself rises monotonically over the whole sweep, from μ_ss_ = 0.414 at v = 10^−4^ m s^−1^ to 0.721 at v = 10 m s^−1^ (Figure 2c). Because the high-rate signal is so noisy, each value is reported with the two uncertainty measures defined in Section 2.3; the error bars in Figure 2c show that the rise is resolved despite the scatter.

The axial displacement of the top platen behaves quite differently (Figure 2b,d). At the slowest rate, the bed contracts slightly, Δh_ss_ = −0.099 mm, so the assembly is still densifying under the applied stress rather than dilating. From v = 10^−3^ m s^−1^ onwards, the bed dilates, and the steady dilation grows with rate to a maximum of Δh_ss_ = 1.02 mm, about one grain diameter, at v = 10^−1^ m s^−1^. Beyond that velocity, it does not continue to grow but falls back to 0.69 mm at 1 m s^−1^ and 0.64 mm at 10 m s^−1^. The two macroscopic responses therefore already part company at this level: the friction increases monotonically across six decades of rate, whereas the volumetric response reverses near v = 10^−1^ m s^−1^. The repeat simulation at that velocity, started from an independently generated packing, reproduces both quantities closely (open squares in Figure 2c,d), so the reversal is not an artefact of a single realisation. Section 3.2 recasts these results in terms of the inertial number, where the consequence of this divergence becomes explicit.

### 3.2. Friction–Solid-Fraction Decoupling

Plotting the steady-state results against the inertial number reveals the central finding of this study: friction and packing do not follow I in the same way (Figure 3). The friction is well described by the standard μ(I) law, μ(I) = μ_s_ + (μ_2_ − μ_s_)/(I_0_/I + 1), with a quasi-static value μ_s_ = 0.43, a high-rate plateau μ_2_ = 0.70, a crossover at I_0_ = 5.5 × 10^−4^, and R^2^ = 0.98 (Figure 3a). Across the full range, the friction follows the μ(I) rheology: nearly constant at low I, rising through the crossover, and saturating at a plateau at high I.

The solid fraction tells a different story (Figure 3b). Starting from φ ≈ 0.577 at the lowest rate, the bulk packing first loosens as the rate increases, reaching a minimum of φ ≈ 0.536 near I ≈ 10^−3^ (v = 0.1 m/s). Beyond this point, however, φ does not keep falling: it recovers to about 0.549 at the highest rate. A single, monotonic φ(I) curve cannot capture this. The best-fit decreasing form leaves clear, systematic residuals on the high-I side, with a degenerate crossover (I_φ_ → 0) that simply reflects the model trying, and failing, to follow a non-monotonic trend. Forcing a monotonic law onto the data is not merely a poorer fit; it is the wrong functional form, because such a law cannot represent a reversal in slope. The residuals are therefore systematic rather than random, changing sign across the minimum, which is the clearest statistical signature that φ carries information not contained in I alone.

Thus, while μ increases monotonically with I, the bulk φ does not follow the same trend. The minimum sits close to the conventional quasi-static–inertial boundary at I ≈ 10^−3^, which suggests that the recovery is tied to the onset of inertial effects. The following sections resolve the flow by height to show where this decoupling comes from. The reproducibility carries over to the rheological plane: the repeat point at I ≈ 10^−3^ (ringed symbols in Figure 3a,b) sits on top of the original in both μ and φ, so the depth of the φ minimum—the quantity on which our interpretation rests—is tightly constrained. Because the inertial number quoted here is a nominal, boundary-driven value, we have quantified how far the local value departs from it (Figure A1). The pressure contributes almost nothing: the grain self-weight adds at most 0.15% to the imposed normal stress over the whole bed, so I(z) is governed by the local shear rate alone. The shear rate does vary, and the resulting I(z) spans 0.5–1.3 times the nominal value at the lowest rate and 0.11–3.6 times it at the highest, where the shear localises. Weighting I(z) by the local shear rate, so that the value represents the material actually deforming, gives an effective inertial number that exceeds the nominal one by a factor of only 1.03 at v = 10^−4^ m s^−1^ and 1.34 at v = 10^−1^ m s^−1^, rising to 2.39 at v = 10 m s^−1^. The nominal I is therefore a good proxy through the quasi-static and dense-flow range, and a fair one at the highest rate. Accordingly, the μ(I) parameters reported below are fitted against nominal I, and we do not claim that the fitted I_0_ is the value a strictly local μ(I) law would return in this heterogeneous bed.

### 3.3. Velocity Profile and Velocity Fluctuation

**Figure 4:**
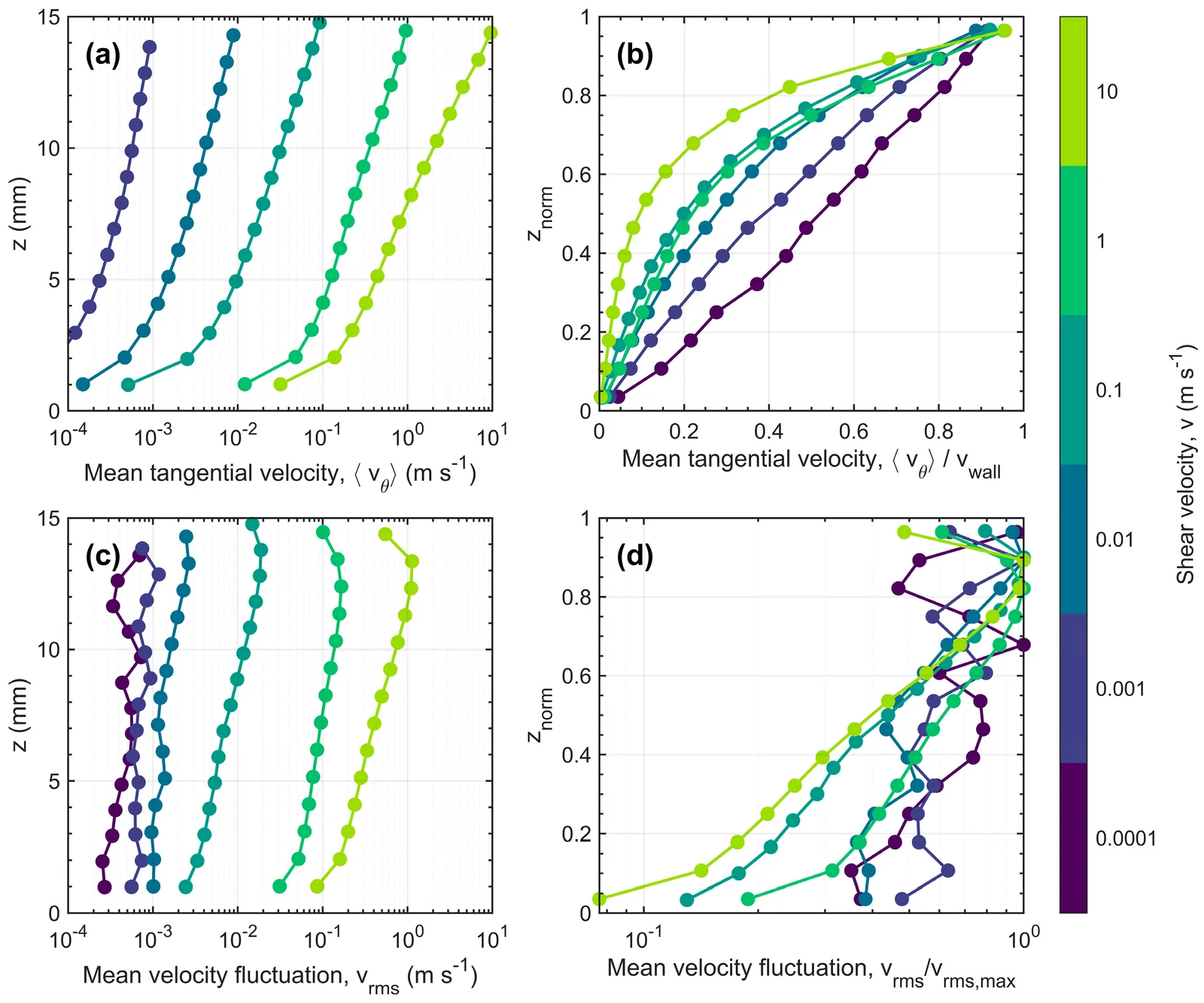
Velocity field versus height for v = 10^−4^–10 m/s. (**a**) Mean tangential velocity (log axis) and (**b**) normalised velocity ⟨v_θ_⟩/v_wall_ versus normalised height; profiles become more concave (more localised) as the rate increases. (**c**) Velocity fluctuation v_rms_ (log axis) and (**d**) normalised fluctuation versus normalised height; fluctuations grow and sharpen toward the top with increasing rate.

The velocity profiles show that shear becomes more localised as the rate increases (Figure 4a,b). At all rates, the tangential velocity rises from near zero at the base to the boundary value at the top [17,40]. When normalised (Figure 4b), however, the profiles change shape systematically. At the lowest rate, the profile is nearly linear, so shear is spread across most of the bed. With increasing shear rate, the velocity profile grows more concave as shear localises into a thinner layer near the driving boundary, and the lower bed becomes increasingly quiescent. Higher rate thus means stronger localization—opposite to the effect of higher confining stress, which spreads shear out. The same exponential-type decay away from the driven boundary is seen in ring-shear experiments on comparable material [41]. The near-wall region that carries most of the shear narrows sharply with rate, from a substantial fraction of the bed at the slowest rate to only a few grain diameters at the fastest. A thinner band means that the same boundary displacement must be accommodated by a smaller mass of grains, which therefore strain, dilate, and agitate more intensely—setting up the strong vertical motion documented below.

**Figure A2:**
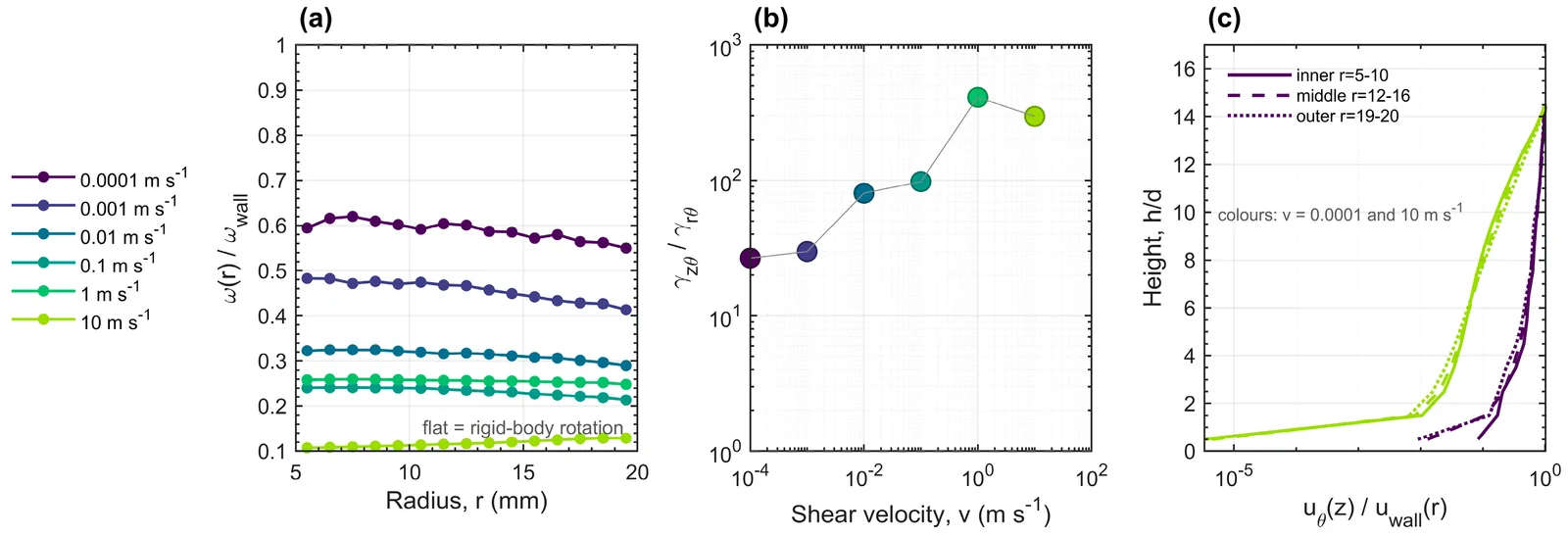
Test for radial gradients in the ring-shear cell. (**a**) Angular velocity ω = uθ/r normalised by its value at the driving wall, against radius, averaged over the mid-height of the bed; a flat profile indicates rigid-body rotation in the horizontal plane and hence negligible in-plane shear. (**b**) Ratio of the vertical to the in-plane-shear rate, the vertical-to-in-plane-shear-rate ratio. (**c**) Normalised vertical velocity profiles measured in inner (r = 5–10 mm), middle (r = 12–16 mm) and outer (r = 19–20 mm) bands, shown for the slowest and fastest cases; line style distinguishes the radial band and colour encodes the shear velocity, dark purple denoting v = 0.0001 m s⁻^1^ and light green v = 10 m s⁻^1^, on the same sequential colour scale used throughout.

The velocity fluctuation v_rms_ follows the same logic (Figure 4c,d). Its magnitude grows by orders of magnitude from the slowest to the fastest case, tracking the rise in granular agitation with rate. Its shape also sharpens: at low rate the normalised fluctuation is fairly uniform with height, whereas at high rate it peaks strongly near the top and decays toward the base, mirroring the localization of the mean flow. Both the mean velocity and its fluctuations confirm that increasing rate pushes deformation and agitation into a shrinking zone near the boundary. The near-uniform low-rate profiles and the sharp, top-peaked profiles at high rate are two ends of the same localization trend, and they bracket the crossover near I ≈ 10^−3^, where the flow begins to concentrate. Because these profiles are measured in a narrow outer band, we checked explicitly that the vertical localization is not a by-product of the radial gradients inherent to a rotating cell (Figure A2). In cylindrical coordinates, the in-plane-shear rate is r ∂(uθ/r)/∂r, so a tangential velocity that grows in proportion to radius carries no shear at all; the relevant test is therefore whether the angular velocity ω = u_θ_/r depends on r, not whether u_θ_ does. It barely does: averaged over the mid-height of the bed, ω varies by only 1.3–6.4% across r = 5–20 mm at every rate, so the horizontal plane is close to rigid-body rotation. Consequently, the vertical shear rate exceeds the in-plane one by a factor of 27 to 411 across the sweep. The localization is also reproduced at every radius: the shear-band thickness measured in inner (r = 5–10 mm), middle (r = 12–16 mm) and outer (r = 19–20 mm) bands is 15/14/15 grain diameters at v = 10^−4^ m s^−1^ and 6/7/8 at v = 10 m s^−1^, and the normalised profiles from the three bands collapse. The structure reported here is thus a genuine vertical localization and not an artefact of sampling near the outer wall.

### 3.4. Solid Volume Fraction

**Figure 5:**
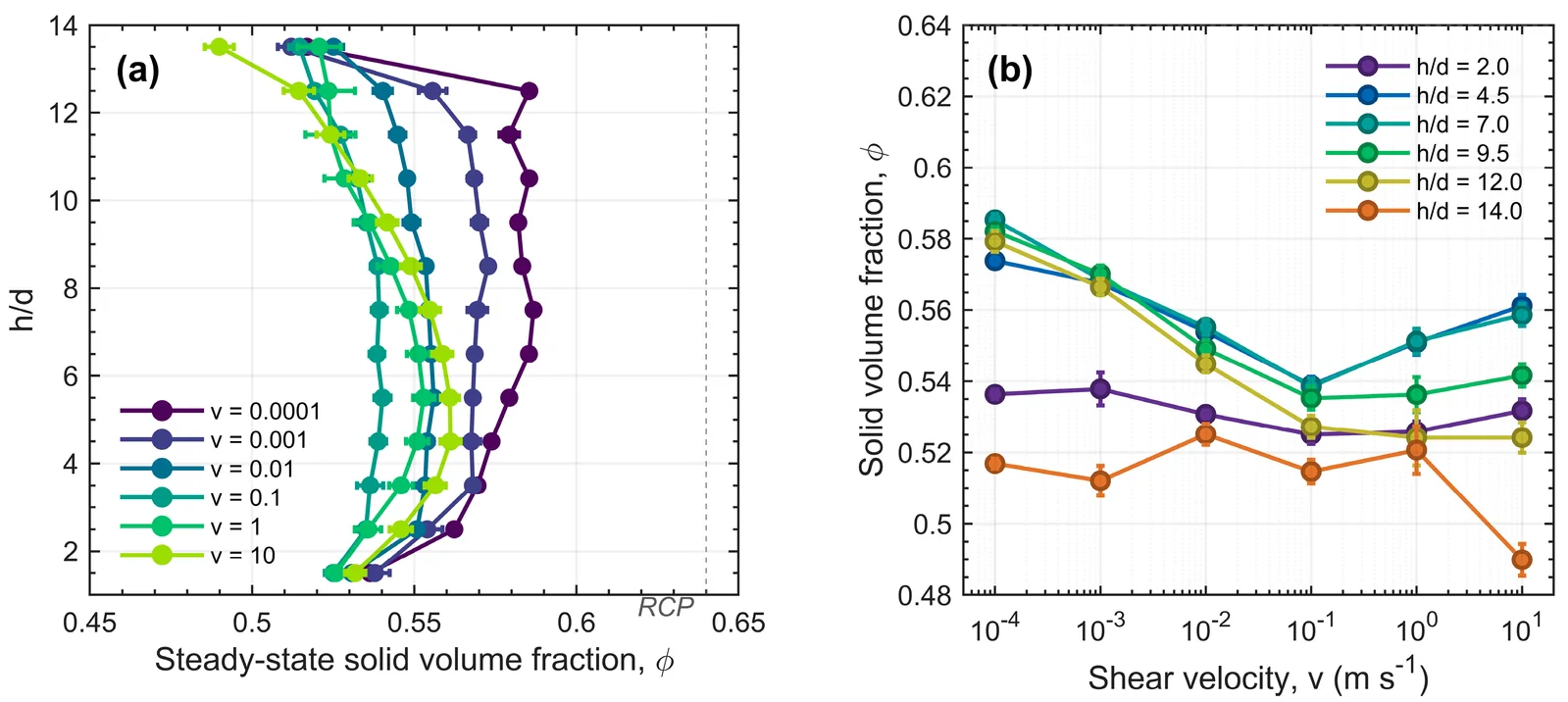
Solid volume fraction. (**a**) Steady-state φ profiles versus normalised height h/d for the six rates (RCP marks random close packing). (**b**) φ versus shear velocity at fixed heights h/d; most layers show a U-shape with a minimum near v = 0.1 m/s, deepest in the mid-bed (h/d ≈ 4.5–7).

In Figure 5a, the dashed line marks φ_RCP_ = 0.64, the standard literature value for the random close packing of monodisperse frictionless spheres; it was not measured in the present simulations and is drawn only as a fixed upper-bound reference. A frictional, sheared and confined assembly cannot reach this bound, because interparticle friction arrests rearrangement well below it and steady shear maintains a critical-state packing that is looser still; the comparison should therefore be read as a reference scale for how far the sheared packing sits below the frictionless ideal, not as a state the material is approaching. The solid-fraction field shows directly how the non-monotonic bulk packing arises (Figure 5). The steady-state profiles (Figure 5a) loosen as the rate increases up to v = 0.1 m/s and partly recover beyond it, with the upper layers always looser than the base. The clearest view comes from plotting φ against shear velocity at fixed heights (Figure 5b). Most layers trace a U-shape: φ falls as the rate increases, reaches a minimum near v = 0.1 m/s, and then recovers at higher rates. The minimum and the recovery are deepest in the mid-bed (h/d ≈ 4.5–7); the near-surface layers (h/d ≈ 9.5–12) dip similarly but recover less, while the basal layer (h/d ≈ 2) stays comparatively dense, with only a weak rate dependence throughout. The contrast between a strongly rate-sensitive upper bed and a nearly rate-insensitive base is the height-resolved fingerprint of the localization seen in the velocity field: only the layers that actually shear can dilate, so the volumetric response is confined to the active zone while the quiescent base holds a fixed, dense packing.

This height-resolved picture explains the bulk trend. Up to v ≈ 0.1 m/s, faster shear drives more dilation everywhere, lowering φ—the standard φ(I) behaviour. Beyond this rate, shear localises into a thin top layer (Figure 4): only that layer keeps dilating, while the now largely passive lower bed re-compacts. The bulk average therefore combines a dilating top with a re-densifying base, and it turns back upward even though the local state at the very top continues to loosen. Overall, the bulk φ recovers not because the material as a whole densifies, but because its denser, more voluminous lower region comes to dominate the average. The depth-average increasingly weights a growing, dense, passive base against a shrinking, dilating active layer, and the balance tips near v ≈ 0.1 m/s. We stress that this redistribution in height is not the whole story: as shown in Section 4.2 and Figure A4, the non-monotonicity persists even when φ is resolved layer by layer and referred to the local inertial number, so the effect cannot be reduced to a mixture of layers each obeying a single-valued φ(I).

### 3.5. Particle Trajectories and Vertical Mixing

**Figure 6:**
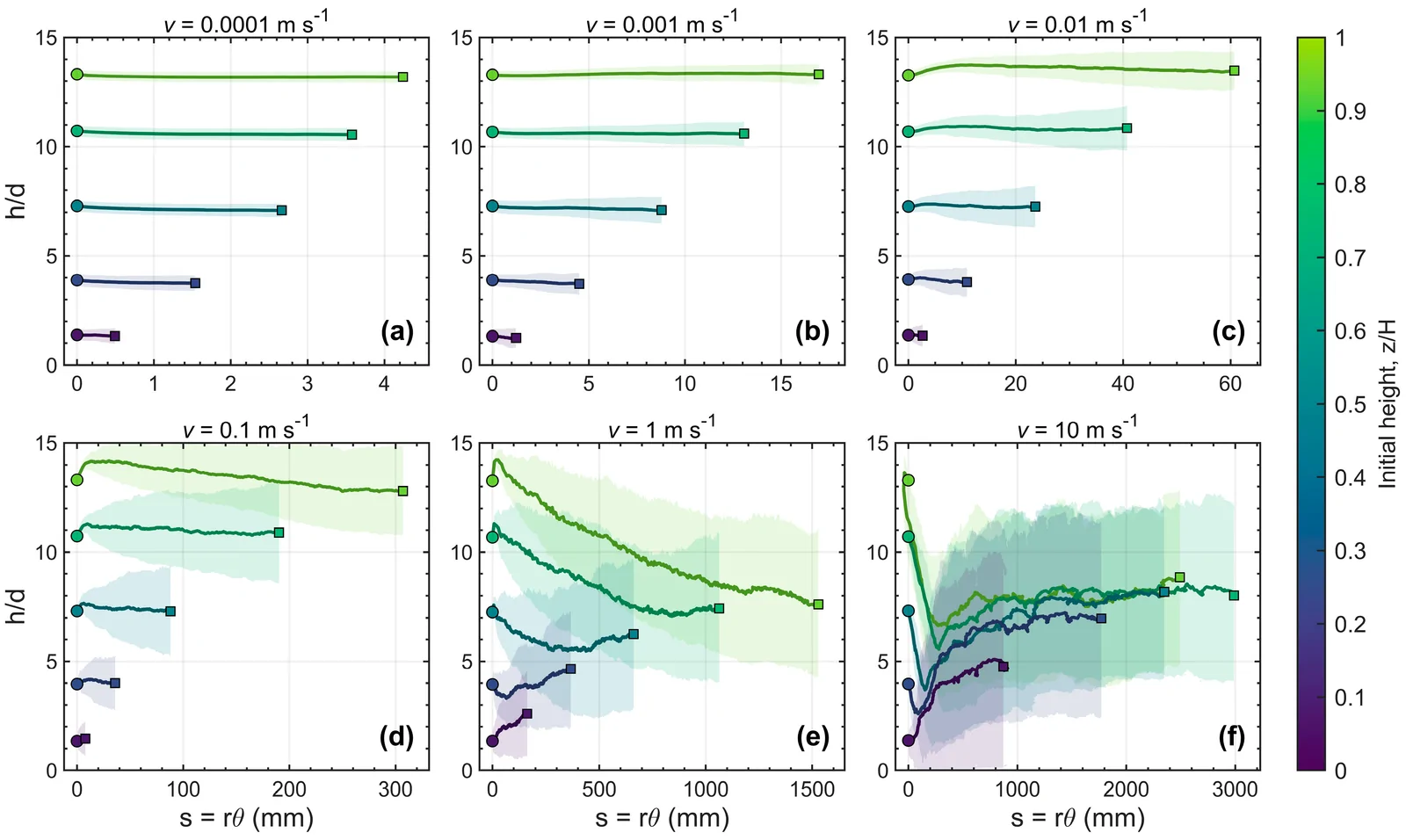
Particle trajectories in the (s, h/d) plane for v = (**a**) 10^−4^, (**b**) 10^−3^, (**c**) 10^−2^, (**d**) 10^−1^, (**e**) 1, and (**f**) 10 m/s; colour encodes initial height z/H. Trajectories are nearly flat at low rate (layers preserved) and show strong cross-layer migration and convergence toward mid-depth at high rate.

**Figure 7:**
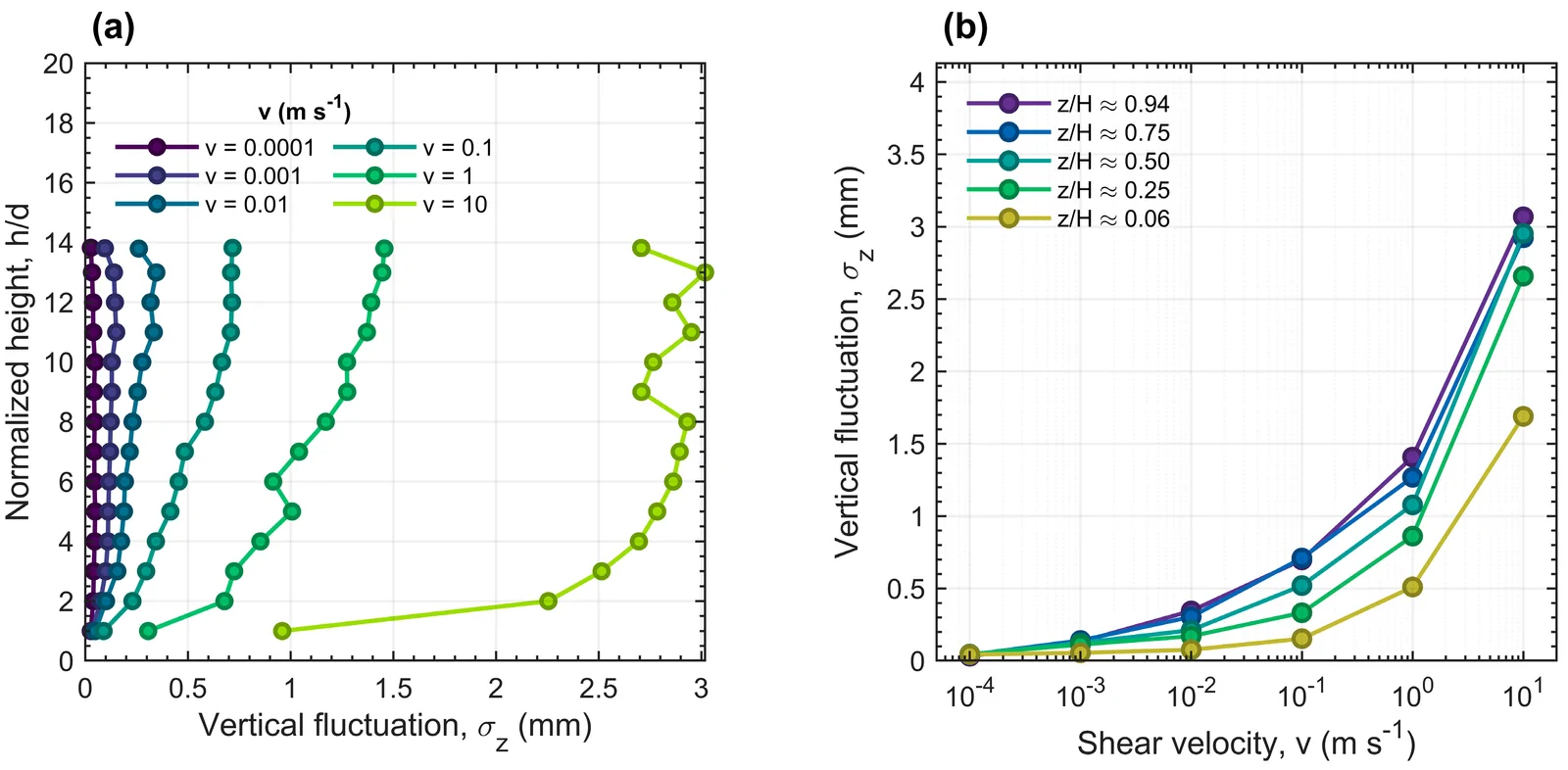
Vertical fluctuation σ_z_. (**a**) σ_z_ versus normalised height for the six rates. (**b**) σ_z_ versus shear velocity at fixed z/H. σ_z_ grows with rate at all heights, reaching about three grain diameters in the upper layers at v = 10 m/s.

Particle tracking shows what drives the re-compaction of the lower bed: vertical mixing that grows sharply with rate (Figure 6 and Figure 7). At low rates (Figure 6a–c), the trajectories are almost flat. Each particle stays close to its starting height, the layers keep their order, and the bed shears like a stack of sliding sheets. At high rates (Figure 6d–f), this layered structure breaks down. Particles make large vertical excursions and migrate across layers; at v = 10 m/s the trajectories from different starting heights converge toward mid-depth, a clear sign of convective-like mixing rather than simple shear. The convergence of trajectories launched from different depths toward a common mid-height band indicates that grains are not merely vibrating in place but are systematically exchanged between layers, so the memory of the initial layering is erased within the sheared region.

The vertical fluctuation σ_z_ quantifies this trend (Figure 7). At every height, σ_z_ grows with rate, and the growth is steepest at high rate (Figure 7b). In the upper layers, it reaches about 3 mm—three grain diameters—at v = 10 m/s, compared with less than 0.1 mm at the slowest rate. Stronger agitation lets grains climb over and past one another and shuffle between layers. This mixing carries loosely packed grains from the active top down into the lower bed and packs the base more tightly, which is the microscopic origin of the φ recovery seen in Figure 5. The threshold character of the effect is notable: σ_z_ stays well below a grain diameter throughout the quasi-static range and only reaches the scale of one diameter—enough for grains to hop out of their cages—once the flow enters the inertial regime, which is precisely where the bulk φ turns around.

### 3.6. Force-Chain Network and Contact-Force Statistics

**Figure 8:**
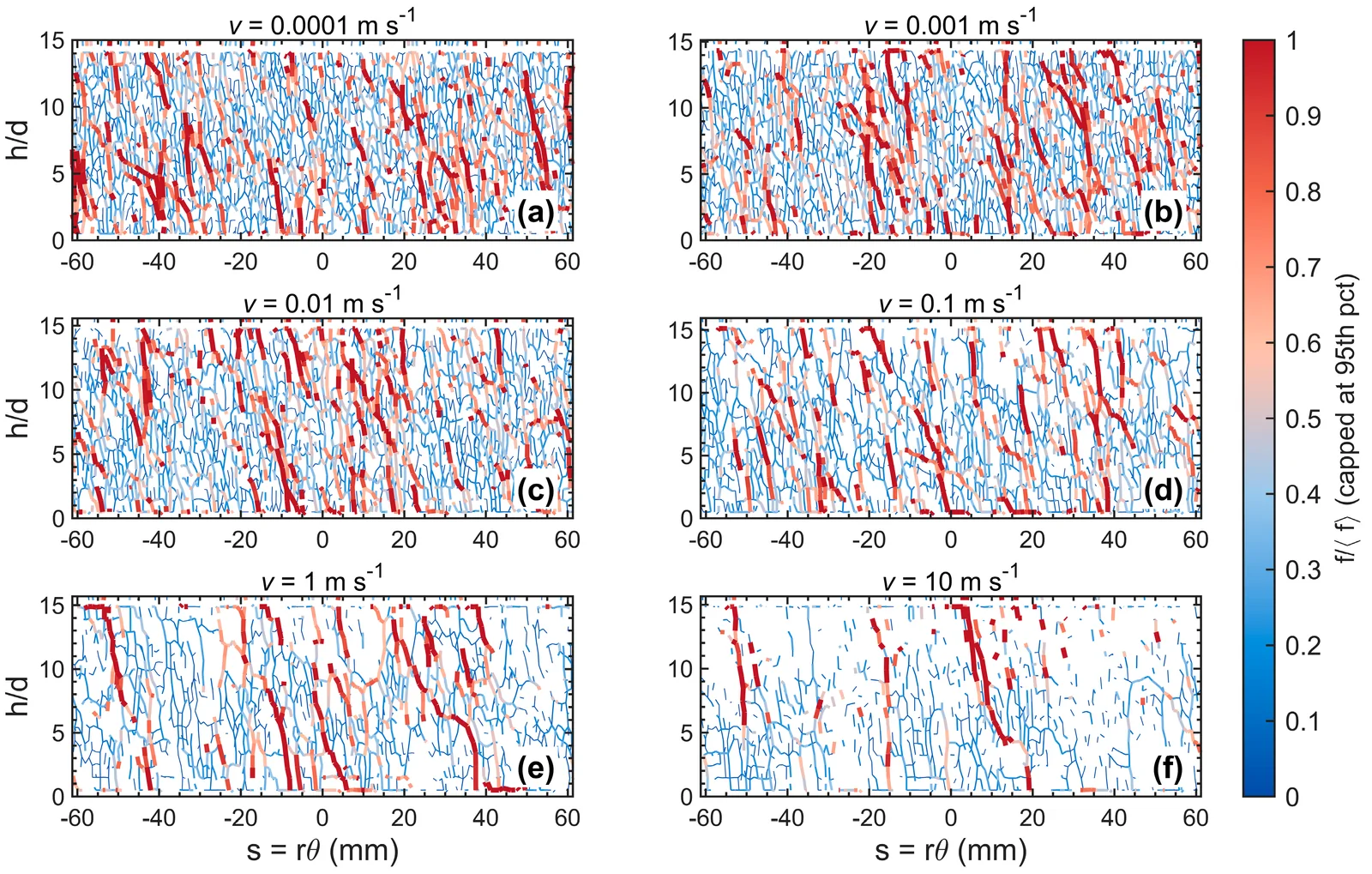
Force-chain networks in the unrolled (s, h/d) plane for v = (**a**) 10^−4^, (**b**) 10^−3^, (**c**) 10^−2^, (**d**) 10^−1^, (**e**) 1, and (**f**) 10 m/s; colour encodes the normalised contact force f/⟨f⟩ (capped at the 95th percentile). The network is dense and uniform at low rate and becomes sparse, more anisotropic, and top-concentrated at high rate; the mean inclination of the strong chains does not increase (Section 3.6 and Figure A3).

**Figure 9:**
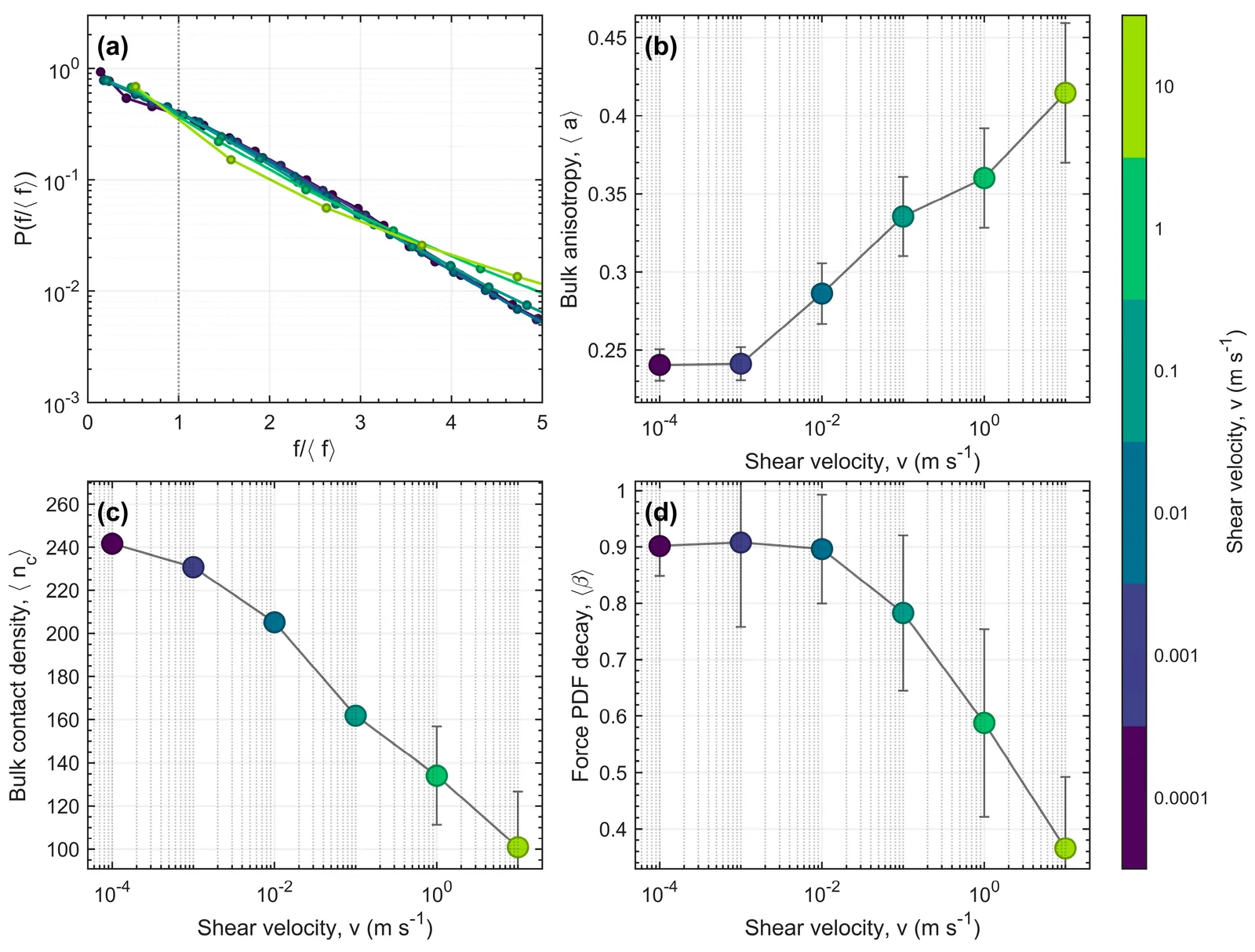
Bulk contact-force statistics and structural trends versus shear velocity (colour encodes v). (**a**) Probability distribution of normalised normal contact forces P(f/⟨f⟩); symbols are data, lines are exponential fits to the tail. (**b**) Bulk fabric anisotropy ⟨a⟩. (**c**) Bulk contact density ⟨n_c_⟩. (**d**) Large-force decay coefficient ⟨β⟩ from the fit P ∝ exp(−*β* f/⟨f⟩). With increasing rate, the anisotropy rises while both the contact density and the decay coefficient fall monotonically, so the network becomes sparser, more anisotropic, and more heterogeneous—carrying its load through fewer, more strongly oriented chains.

**Figure A3:**
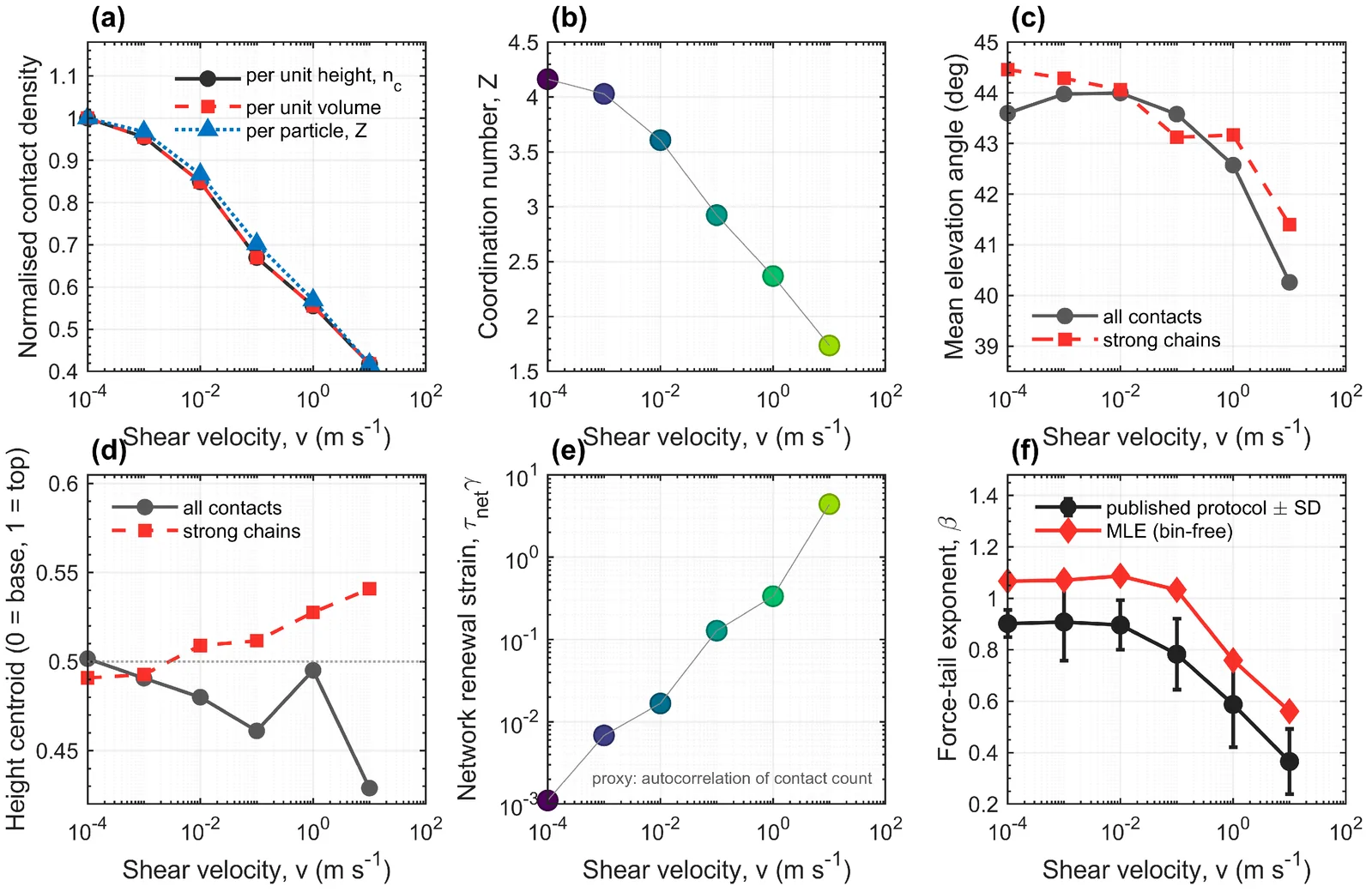
Quantitative characterisation of the contact network. (**a**) Contact density under three normalisations—per unit height, per unit volume, and per particle (coordination number Z)—each divided by its value at the lowest rate. (**b**) Coordination number Z. (**c**) Mean elevation angle of the branch vector above the shear plane, for all contacts and for the strong chains (F ≥ 1.5⟨F⟩). (**d**) Height centroid of the contact population (0 = base, 1 = top of the band). (**e**) Shear strain required to renew the contact network, used as a proxy for network persistence. (**f**) Force-tail exponent β from the published per-snapshot histogram protocol (with its scatter across snapshots), from a binning-free maximum-likelihood estimator, and from pooled histogram fits at several tail thresholds.

The contact network reorganises steadily as the rate increases (Figure 8 and Figure 9). At low rate, the force network is dense and rather uniform, with strong chains spread through the full height and aligned close to the shear plane (Figure 8a; [42,43]). As inertia grows, the network becomes sparse and patchy, and the strong chains migrate toward the top of the bed (Figure 8f), so the bed carries its load through fewer and more strongly oriented columns. We have quantified each of these descriptions (Figure A3), and they are not all borne out. The vertical concentration is confirmed: the height centroid of the strong-chain population (0 = base, 1 = top of the band) moves upward from 0.491 to 0.541, and the share of strong contacts lying in the upper third of the bed grows from 0.31 to 0.39. Notably, the contact population as a whole moves the other way, its centroid falling from 0.502 to 0.429, so it is specifically the load-bearing chains that migrate toward the driving boundary while the bulk of the contacts settles into the re-compacting base. Network persistence cannot be measured directly, because the exported contact data carry no contact identifiers; as a proxy, we use the integrated autocorrelation time of the contact count, expressed as the shear strain over which the network renews itself. This falls from 0.0011 to 4.40, so at the highest rate the network is rebuilt within a strain of order unity—a turnover consistent with the buckling-mediated failure of force chains described by Tordesillas [44]—and with the large friction fluctuations of Figure 2a, whose relative amplitude grows from 0.018 to 0.68 over the same range. The orientation, by contrast, does not support our earlier wording: the mean elevation of the strong-chain branch vectors above the shear plane is 44.5° at the lowest rate and 41.4° at the highest, so the chains do not become steeper. What does change is the strength of the alignment rather than its direction—the fabric anisotropy rises from 0.24 to 0.40 (Figure 9b)—and we have amended the text accordingly. This reorganisation is also spatially graded: the fabric anisotropy, the contact-force gradient, and the strong-chain fraction all intensify toward the top as the flow localises, mirroring the velocity and solid-fraction fields.

The bulk statistics make the trend quantitative (Figure 9). The distribution of normal contact forces P(f/⟨f⟩) keeps the form familiar from dense packings at every rate—a broad peak below the mean from the many weak contacts, and a near-exponential tail carried by the strong chains (Figure 9a; [42,45]). What changes is the weight of that tail: fitting P ∝ exp(−β f/⟨f⟩) over the tail f/⟨f⟩ ≥ 1, using a probability-density histogram of max(15, √N) bins and fitting each snapshot separately before averaging over snapshots, the decay coefficient β stays near 0.90 through the quasi-static range (v ≤ 10^−2^ m/s) and then falls steeply to 0.37 at the highest rate (Figure 9d). Because such tail fits are known to depend on the threshold and on the binning, we also evaluated β with a binning-free maximum-likelihood estimator, β = 1/(⟨f⟩_tail_ − 1), which is the efficient estimator for an exponential tail and requires no histogram at all; it gives 1.07 and 0.56 at the two ends of the range. The two estimators differ in absolute value, the histogram fit lying lower because the tail is sampled sparsely once the network becomes dilute, but both show β constant through the quasi-static range and then falling once the flow becomes inertial, by a factor of 2.5 and 1.9 respectively. Across tail thresholds from f/⟨f⟩ = 0.5 to 2, the fitted values change by less than the difference between the two estimators, so the broadening of the force distribution with rate is robust to how the tail is defined and fitted; the scatter of β across individual snapshots is shown in Figure A3f, so an increasing share of the load concentrates in a few strong, transient chains and the network grows markedly more heterogeneous [21]. The bulk fabric anisotropy ⟨a⟩ follows suit, low and rate-independent in the quasi-static range (≈0.24) and rising monotonically to about 0.41 at the highest rate (Figure 9b). Most importantly, the bulk contact density ⟨n_c_⟩ falls monotonically from about 242 to 101 per millimetre across the sweep, without reversal—including the interval where the bulk solid fraction recovers (Figure 9c). Because a contact count per unit height could in principle change simply through the bed height or the number of layers, we repeated the measurement under two further normalisations: contacts per unit volume, which falls from 1.973 to 0.824 mm^−3^, and the coordination number Z = 2N_c_/N_p_, which falls from 4.16 to 1.74. All three fall by the same factor, 0.42, over the sweep, while the bed height itself changes by only a few per cent, so the loss of connectivity is a real reorganisation of the contact network and not an artefact of the normalisation. The connectivity of the network thus diverges from φ exactly where the φ(I) relation breaks down: the load-bearing structure loses contacts and grows more anisotropic and heterogeneous in a single monotonic progression that mirrors the friction, identifying contact connectivity, rather than the volume the grains fill, as the structural quantity that tracks the macroscopic response.

## 4. Discussion

### 4.1. A Coherent Rate-Dependent Sequence

Taken together, the results trace a single, ordered sequence as the shear rate climbs through six decades. At the lowest rates (quasi-static, I → 0), shear spreads through almost the whole bed, the velocity profile is close to linear, the force network is dense and uniform, the contact density is high, and the packing is dense. As the rate increases, the shear band narrows toward the driving boundary, dilation strengthens in the classical Reynolds sense—shear of a dense packing requires the grains to ride over one another, so that deformation and volume change are kinematically coupled [46,47]—fluctuations grow, and the bulk solid fraction falls, which is the standard μ(I)/φ(I) behaviour of the dense flow regime. Near v ≈ 0.1 m/s (I ≈ 10^−3^), the bulk packing reaches its loosest state. Beyond this rate, in the inertial regime, vertical agitation becomes strong enough (σ_z_ up to about 3d) to drive cross-layer mixing: deformation concentrates in a thin top layer while the lower bed re-compacts, so the bulk φ recovers [7,48]. Throughout, the force network grows sparser and more anisotropic; its load-bearing chains concentrate toward the driving boundary and renew themselves after ever smaller strains, and the contact density falls. The minimum in bulk φ marks the changeover from dilation-controlled loosening to mixing-controlled re-compaction, and it coincides with the classical quasi-static–inertial boundary. The width of the crossover is itself informative: friction, dilation, localization, agitation, and fabric all begin to change most rapidly in the same narrow band of I around 10^−3^, which argues that a single physical transition—the onset of inertial, collisional transport—drives the whole set of responses, rather than several unrelated effects happening to coincide.

It is worth placing this non-monotonic response beside a body of work on vibrated granular flows, in which a comparably non-monotonic behaviour has been reported and traced to the level of agitation. Gnoli et al. [49] showed that the effective viscosity of a dense granular medium can be tuned, and made non-monotonic, by externally imposed vibration; Clark et al. [50] found that vibration weakens the friction of a granular flow; and Plati et al. [51] showed that the non-monotonic rheology of dense vibrated flows is controlled by the granular temperature. The parallel with the present results is close, and we think it instructive. In those systems the agitation is supplied externally by the vibrating boundary, whereas here it is generated by the shear itself, growing with rate until the vertical velocity fluctuations reach σ_z_ ≈ 3d and drive cross-layer mixing. In both cases it is the agitation, rather than the imposed shear rate alone, that sets the packing state, so that a description based on I alone becomes incomplete. The granular temperature we measure (Section 3.3) is the same quantity that Plati et al. [51] identify as the controlling variable, which suggests that the rate-driven and vibration-driven cases are two routes to a single mechanism. We regard this correspondence as support for reading the present decoupling as agitation-controlled rather than as a peculiarity of ring-shear geometry.

This sequence is the rate-controlled counterpart to the better-known stress-controlled one. Raising the confining stress densifies contacts and broadens the shear zone; raising the shear rate does the opposite, sharpening localization [22]. Both can be read as motion along the same inertial-number axis—lower I for higher stress, higher I for higher rate—which places the present results within the common μ(I) picture while highlighting a response that this picture does not anticipate.

### 4.2. Why Friction and Packing Decouple

The decoupling of μ and φ follows naturally once the flow is seen as stratified rather than uniform. The macroscopic friction is set at the driving boundary, where the shear stress is transmitted; it reflects the state of the active layer that actually carries the deformation. As the rate rises, that layer behaves as the μ(I) law predicts, and μ_ss_ follows I closely.

**Figure A4:**
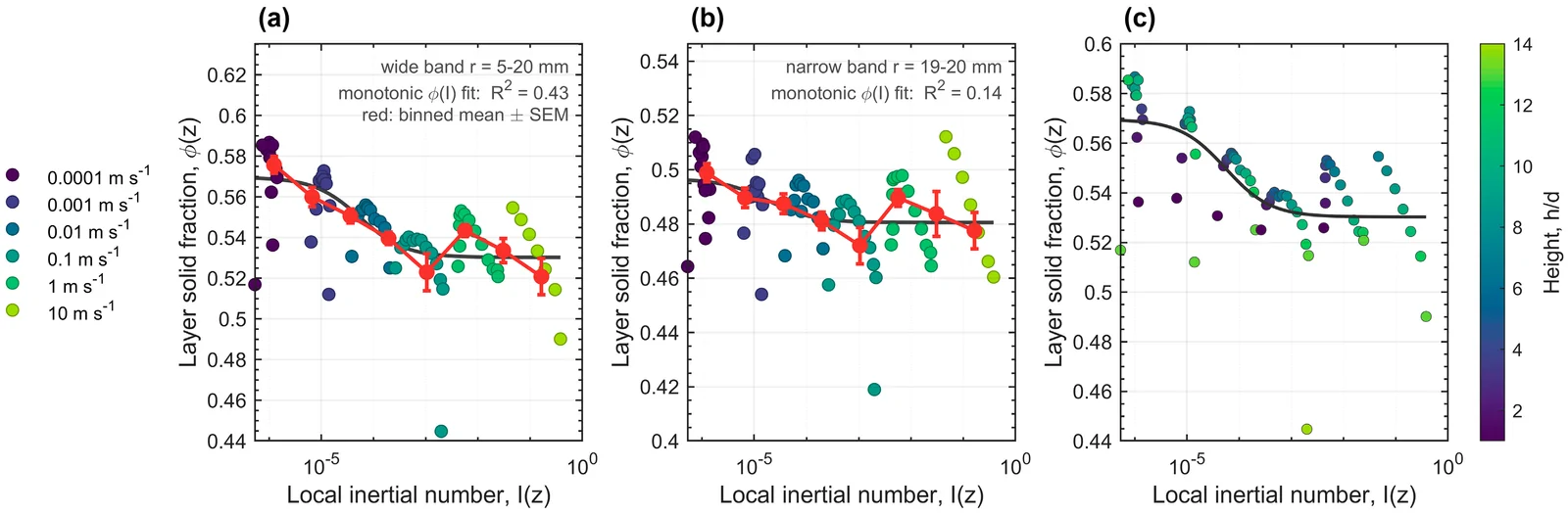
Layer-resolved solid fraction against the local inertial number, for layers inside the shear band. (**a**) φ evaluated on the wide band (r = 5–20 mm), the sampling used for the profiles of Figure 5; the black curve is the best-fitting monotonic φ(I) law and the red curve is the mean of the layer data in logarithmic I bins, with error bars showing the standard error. (**b**) The same test using the narrow band (r = 19–20 mm), i.e., exactly the material for which the shear rate is measured. (**c**) The wide-band data coloured by height. The binned means retain a minimum and a recovery, so the non-monotonicity is not removed by resolving φ layer by layer.

The bulk solid fraction, by contrast, is an average over the whole column, and the column is not in a single state. Its top dilates and shears while its base stays dense and nearly still, and the split between the two shifts with rate. Below the φ minimum, faster shear loosens the bed as a whole. Above it, localization and mixing redistribute material in height: the active top thins and loosens further, but the passive base re-densifies and, being larger and more populated, pulls the average back up. The bulk φ thus stops being a clean function of I—not because the local rheology breaks down, but because a single number can no longer represent a strongly graded field. This is the same difficulty that motivates nonlocal rheology, where a local μ(I) closure is known to fail once strain-rate gradients become sharp [11,13,14]. Here the failure appears specifically in the volumetric response, while the frictional response, tied to the active layer, survives. It is worth stressing that nothing in this account requires the local μ(I) or φ(I) relations to be wrong; each layer may obey them faithfully. We therefore tested directly whether the effect is merely an artefact of depth-averaging, by recomputing the solid-fraction layer by layer and plotting it against the local inertial number I(z) resolved in the same layers (Figure A4). It is not. The layer-resolved data do not collapse onto a single monotonic φ(I) curve—the best-fitting monotonic law explains only R^2^ = 0.43 of their variance—and, more tellingly, the binned layer data retain a minimum at I(z) ≈ 1.0 × 10^−3^, followed by a recovery of Δφ = 0.021, or 2.1 times the combined standard error. That minimum falls at the same inertial number at which the bulk φ is loosest. The decoupling therefore survives layer-resolved reporting: the volumetric response is not a single-valued function of the local I, whereas the friction is. This is precisely the situation that motivates nonlocal rheology, in which a local closure fails once strain-rate gradients become sharp, and it shows that the effect reported here is a genuine property of the flow rather than a consequence of how φ is averaged. This makes the effect both robust—it must appear whenever shear localises strongly—and easy to overlook, because bulk φ is often the only volumetric quantity that experiments can readily access.

### 4.3. Contact Density as a Robust Order Parameter

If bulk φ is unreliable in this regime, what should replace it? Our results point to the contact density n_c_. Unlike φ, n_c_ decreases monotonically with rate (Figure 9c) and so stays in step with the friction. The reason is physical: n_c_ counts load-bearing contacts directly, whereas φ counts the volume that grains occupy. When mixing repacks the lower bed, φ can rise even though grains there form fewer lasting contacts, because transient, agitated packing fills space without building a stable network. The friction, which depends on how many contacts carry load and how they are oriented, follows n_c_ and the fabric rather than φ. This distinction—between how much space grains fill and how well they are connected—has long been recognised in studies of fabric and force anisotropy [37,39,45,52]. We should be explicit about the status of this claim. What the simulations establish is a correspondence: over the range tested, n_c_ and μ vary monotonically together while φ does not. That correspondence is descriptive, and the present data do not by themselves demonstrate that connectivity causes the frictional response. A causal argument would require independent control of n_c_ at fixed I, for example by varying interparticle friction or polydispersity at constant inertial number, which we have not attempted here. What can be said on mechanical grounds is that the shear stress is transmitted through the contact network, so a state variable that counts load-bearing contacts is a more natural carrier of the frictional response than one that counts occupied volume; on that basis we propose n_c_ as the better state variable for these conditions, and identify the causal test as a target for future work. Treating contact density, or more generally the fabric, as a coupled internal variable alongside friction would let continuum models reproduce the present behaviour, which a φ(I) closure alone cannot [11,16]. The bulk force statistics of Figure 9 reinforce this reading. The steady fall of the contact density, the simultaneous broadening of the force distribution (smaller β), and the growth of the fabric anisotropy all vary monotonically with rate, in step with the friction, whereas bulk φ does not. Connectivity and force heterogeneity, not the volume fraction, are what the friction actually follows, which is why a state description built on the fabric succeeds where one built on packing fraction fails.

### 4.4. Implications and Limitations

The practical message is that the bulk packing fraction should be used with care as a state variable in rate-dependent dense flows. Where shear localises—near retaining structures, along shear bands, in fault gouge, or at the base of a flowing layer—an apparent solid fraction averaged over depth can move opposite to the local trend and give a misleading read on the material state [17,53]. The friction and the contact density are steadier guides. The clean μ(I) fit obtained here also provides calibration values (μ_s_ = 0.43, μ_2_ = 0.70, I_0_ = 5.5 × 10^−4^) for glass beads at 100 kPa, while the non-monotonic φ and the σ_z_ and n_c_ profiles offer specific targets for models that aim to capture rate effects beyond the local μ(I) closure [11,23]. The reproducibility of the v = 0.1 m/s case, shown in Figure 2 and Figure 3, bears on this point as well: because the loosest state is the most delicate part of the whole sweep, its independent recovery gives confidence that the reported φ minimum—and hence the calibration of any rate-dependent closure against it—is not an artefact of a single realisation.

Two limitations bound these conclusions. First, the grains are monodisperse, non-cohesive, non-breaking spheres. Shape, size spread, cohesion, and breakage all affect dilatancy and fabric evolution and could shift the rate of the φ minimum or the strength of the recovery, even if the qualitative ordering survives [44,54,55,56]. Second, the study fixes the confining stress at 100 kPa and uses a single bed height; mapping the φ minimum and the n_c_ trend across stress and height would test how general the decoupling is. This bears directly on how far the present numbers can be scaled. The inertial number is constructed to collapse rate and stress, so the qualitative sequence reported here—dilation first, then localization and mixing-driven re-compaction—should recur in any system that localises, and the position of the φ minimum near I ≈ 10^−3^ coincides with the classical quasi-static-to-inertial crossover rather than with anything specific to these parameters. The fitted constants are another matter. I_0_ is not a universal number: it is known to depend on interparticle friction, on grain shape and polydispersity, and on the stiffness of the grains, and reported values span several orders of magnitude across materials and geometries [5,6,10], as quantified below. The values given here (μ_s_ = 0.43, μ_2_ = 0.70, I_0_ = 5.5 × 10^−4^) should therefore be treated as a calibration for monodisperse spherical glass beads at 100 kPa in this geometry, not as material-independent constants. The same caution applies to the parameter space more broadly: cohesion, polydispersity, non-spherical shape, a different interparticle friction, and softer grains would each alter dilatancy and fabric evolution, and so could shift the rate at which φ is loosest and the depth of the recovery, even if the ordering of events survives [54,55,56]. We expect the decoupling itself to be robust, because it requires only that shear localises while the passive region re-compacts, but its quantitative signature is material-dependent and each of these factors deserves a dedicated study.

Because I_0_ is the parameter on which any transfer of these results to another material or geometry depends, it deserves to be examined rather than simply quoted. Fitting the six steady-state points returns I_0_ = (5.5 ± 2.1) × 10^−4^, the interval being the standard error of the non-linear least-squares fit; constraining I_0_ to 10^−3^ instead lowers R^2^ only from 0.983 to 0.973, and any value between about 3 × 10^−4^ and 10^−3^ fits the data with R^2^ > 0.97. Six points therefore locate the crossover to within a factor of two or three, and no better, which is itself a caution against treating I_0_ as a sharply defined material constant. The comparison with published values is more striking. For the same functional form, free-surface flows of glass beads of comparable diameter are described with I_0_ ≈ 0.3 [10], nearly three orders of magnitude above the value found here, while compilations of plane-shear data place the quasi-static-to-inertial crossover nearer I ≈ 10^−3^–10^−2^ [5,6]. This is not a disagreement about the material but about what the fit is asked to describe. In the confined cell, the friction is not flat in the quasi-static range: μ rises by 18% between I ≈ 10^−6^ and 10^−4^, the slow, roughly logarithmic rate dependence long documented for confined sheared packings [21], whereas inclined-plane and free-surface experiments seldom reach below I ≈ 10^−2^ and so do not sample it. A fit that spans those extra decades necessarily places its half-rise point low. I_0_ therefore encodes the loading configuration and the range of I explored as well as the grain properties, and annular shear covering both regimes has likewise been found to require geometry-specific parameters [13]. It is best read as a fitted crossover for a given cell and rate range, not as a material constant.

What does appear transferable is not I_0_ itself but where the packing minimum falls relative to it. The loosest state occurs at I = 1.05 × 10^−3^, about twice the fitted I_0_, and the recovery begins immediately beyond. Both quantities mark the same physical event—the rate at which grain inertia begins to compete with the confining pressure—so the minimum should track I_0_ rather than sit at a fixed absolute I. That is a concrete prediction: in a material with a larger I_0_, for instance one with rougher or more angular grains, the φ minimum should move to a correspondingly higher rate while the ratio remains of order unity. Confirming it requires repeating the sweep for a second material and a second confining stress, which lies outside the present study but is the natural next step, and it is the form in which we would expect these results to generalise. The same reasoning applies to μ_s_ and μ_2_, which set the amplitude of the frictional rise but not the rate at which it occurs, and which we would likewise expect to shift with interparticle friction and grain shape while leaving the decoupling itself intact.

A related question is whether the effect could be confirmed experimentally. The bulk quantities involved—the friction and the height of the loading platen—are precisely what a laboratory ring-shear apparatus measures, so the non-monotonic bulk φ is directly testable and, to our knowledge, has not been sought over six decades of rate in a single device. What is far harder experimentally is the interior evidence that explains it: the height-resolved velocity, solid fraction, vertical mixing and contact network. Optical and X-ray methods can reach some of these in index-matched or small assemblies, but resolving the contact network at 100 kPa while sweeping six decades of shear rate is not currently practical. That asymmetry—bulk response measurable, not internal cause—is what motivates the computational approach adopted here. We regard the clear separation of the frictional and volumetric responses, and the identification of contact density as the robust order parameter, as the main contributions of this study.

## 5. Conclusions

We used three-dimensional DEM simulations of annular shear to follow a dense glass-bead assembly at a fixed normal stress of 100 kPa, while the shear velocity was varied over six orders of magnitude (10^−4^ to 10 m/s; I ≈ 10^−6^ to 10^−1^). The main findings are as follows.

(1) The macroscopic friction follows the μ(I) law closely (μ_s_ = 0.43, μ_2_ = 0.70, I_0_ = 5.5 × 10^−4^, R^2^ = 0.98) and rises monotonically with rate, whereas the bulk solid fraction is non-monotonic—falling to a minimum near v = 0.1 m/s (I ≈ 10^−3^) and recovering at higher rates—so a single φ(I) curve fits poorly. Friction and packing therefore decouple in their dependence on the inertial number.
(2) Height-resolved fields explain the decoupling. As the rate increases, shear localises into a thin layer near the driving boundary, and strong vertical agitation (σ_z_ up to about three grain diameters) drives cross-layer mixing that re-compacts the lower bed. The bulk φ averages a dilating top with a re-densifying base and recovers, even though the active layer keeps loosening.
(3) The contact density n_c_ decreases monotonically with rate and stays in step with the friction, which makes it a more reliable structural order parameter than bulk φ in this regime. The force network also grows sparser and more anisotropic with rate, while the mean inclination of its strong chains changes little (44.5° to 41.4°).

These results show that the frictional and volumetric responses of a dense granular flow can depend on shear rate in different terms, and that the bulk solid fraction should be treated with caution as a state variable when the flow is localised. The friction and the contact density provide steadier descriptions, and the profiles reported here give quantitative targets for rate-dependent constitutive models.

## Figures and Tables

**Table 1:** DEM model parameters.

Parameter	Symbol	Value
Particle density	*ρ*	2500 kg/m^3^
Shear modulus	*G*	30 GPa
Poisson’s ratio	*ν*	0.2
Particle diameter	*d*	1 mm
Restitution coefficient	*e*	0.938
Particle–particle sliding friction	*μ_p_*	0.9
Particle–particle rolling friction	*μ_r_*	0.1
Particle–wall sliding friction	*μ_pw_*	0.04
Particle–wall rolling friction	*μ_rw_*	0.04
Initial bed height	*H*	15 mm
Outer radius	*R*	20 mm
Normal stress	*σ*	100 kPa
Time step	*Δt*	0.2 t_R_

**Table 2:** Steady-state values and their uncertainties. SD is the standard deviation of the raw time series over the averaging window; SEM_eff_ is the standard error of the mean corrected for temporal correlation through the integrated autocorrelation time τ_int_ and the effective sample size N_eff_. The last two columns give the duration of the averaging window and the shear strain accumulated within it.

v (m s^−1^)	I (Nominal)	μ_ss_	SD (μ)	SEM_eff_ (μ)	τ_int_ (s)	N_eff_	Window (s)	Strain
0.0001	1.05 × 10^−6^	0.4140	0.0073	0.0020	1.033	13.6	28.0	0.19
0.001	1.05 × 10^−5^	0.4294	0.0141	0.0066	1.270	4.6	11.7	0.78
0.01	1.05 × 10^−4^	0.4901	0.0122	0.0022	0.071	31.7	4.5	3.00
0.1	1.05 × 10^−3^	0.5994	0.0266	0.0020	0.008	174.2	2.7	18.00
1	1.05 × 10^−2^	0.6701	0.3618	0.0219	0.005	274.0	2.7	180.00
10	1.05 × 10^−1^	0.7212	0.4909	0.0328	0.006	224.1	2.7	1800.00

## Data Availability

The original contributions presented in this study are included in the article. Further inquiries can be directed to the corresponding author.
